# Planetary health as a main topic for the qualification in digital teaching – a project report

**DOI:** 10.3205/zma001617

**Published:** 2023-05-15

**Authors:** Kristina Flägel, Mattis Manke, Katharina Zimmermann, Stefan Wagener, Saskia Veronika Pante, Mirijam Lehmann, Sabine C. Herpertz, Martin R. Fischer, Jana Jünger

**Affiliations:** 1 Universitätsklinikum Schleswig-Holstein, Campus Lübeck, Institut für Allgemeinmedizin, Lübeck, Germany; 2 Bundesvertretung der Medizinstudierenden in Deutschland e. V. (bvmd), Berlin, Germany; 3 Universität Heidelberg, Medizinische Fakultät, Studiengang Master of Medical Education (MME), Heidelberg, Germany; 4 Universitätsklinikum Heidelberg, Klinik für Allgemeine Psychiatrie, Heidelberg, Germany; 5 LMU München, LMU Klinikum, Institut für Didaktik und Ausbildungsforschung in der Medizin, München, Germany; 6 Institut für Kommunikations- und Prüfungsforschung gGmbH, Heidelberg, Germany

**Keywords:** planetary health, medical education, curriculum, supervision, scholar, role of multiplicator

## Abstract

**Aim:**

To do justice to the need for planetary health in medical education, these are the aims of the online elective course “Planetary Health in Medical Education” (ME elective):

1. Enable students to plan and realize their own course sessions on planetary health;

2. Encourage communication among university medical faculties regarding planetary health in medical education;

3. Reinforce competency in digital teaching and amplify the expert role as multiplicator among students pursuing a Master's degree in Medicinal Education (MME).

**Method:**

The development of the ME elective followed Kern's six-step approach to curriculum development by means of cooperation between the German Medical Students' Association (Bundesvertretung der Medizinstudierenden in Deutschland, abbreviated as bvmd), and the MME study program. Based on general and specific needs analyses, core learning objectives regarding planetary health, medical education and digital education were identified in the National Catalogue of Learning Objectives in Undergraduate Medical Education (NKLM) and the MME study program and relevant teaching methods were selected.

**Results:**

The ME elective, consisting of two contact hours per week per semester, was established at 13 medical schools as a four-phase course:

1. Introduction to medical education using examples from planetary health;

2. Lesson planning on a topic in planetary health under the supervision of MME students;

3. Course sessions held by the undergraduate students; and

4. Networking with the MME study program through participation in digital courses on planetary health and the pilot OSCE on planetary health.

A total of 24 students attended the pilot in the 2022 summer semester.

**Conclusion:**

The topic of planetary health combines interests that span many subjects and semester levels. As a collaborative, interdisciplinary and interprofessional subject, it lends itself to training students in a trans-institutional elective course to become multiplicators.

## Zusammenfassung

**Zielsetzung:**

Um den Anforderungen der Planetaren Gesundheit in der medizinischen Ausbildung gerecht zu werden, ist es Ziel des Online-Wahlfachs „Medizinische Ausbildung der Planetaren Gesundheit“ (WFMA)

1. Studierende zu befähigen, eigene Lehrveranstaltungen zur Planetaren Gesundheit zu planen und durchzuführen,

2. den fakultätsübergreifenden Austausch zu Planetarer Gesundheit in der medizinischen Ausbildung zu stärken,

3. die Kompetenzen in digitaler Lehre sowie die Expert*innenrolle als Multiplikator*in der Studierenden des Masterstudiengangs Medical Education (MME) zu vertiefen.

**Methodik:**

Die Entwicklung des WFMA folgte den sechs Schritten der Curriculumsentwicklung nach Kern als Kooperation zwischen der Bundesvertretung der Medizinstudierenden in Deutschland (bvmd) und dem MME. Aufbauend auf genereller und gezielter Bedarfsanalyse wurden übergeordnete Lernziele zu Planetarer Gesundheit, medizinischer Ausbildung und digitaler Lehre aus dem NKLM und dem MME identifiziert, und entsprechende Lehrmethoden ausgewählt.

**Ergebnisse:**

Das WFMA mit zwei Semesterwochenstunden wurde an 13 Fakultäten mit vier Phasen etabliert:

1. Einführung zur medizinischen Ausbildung anhand von Beispielen aus dem Bereich Planetare Gesundheit,

2. Veranstaltungsplanung zu einem Thema der Planetaren Gesundheit unter Supervision von MME-Studierenden,

3. Veranstaltungsdurchführung durch die Studierenden sowie

4. Vernetzung mit dem MME durch Teilhabe an digitalen Lehrveranstaltungen zur Planetaren Gesundheit und dem Probe-OSCE „Planetare Gesundheit“.

24 Studierende nahmen an der Pilotierung im Sommersemester 2022 teil.

**Schlussfolgerung:**

Das Thema Planetare Gesundheit vereint fächer- und semesterübergreifende Interessen. Als kollaborative, interdisziplinäre und interprofessionelle Aufgabe ist es geeignet, Studierende in einem fakultätsübergreifenden Wahlfach gemeinsam zu Multiplikator*innen auszubilden.

## 1. Introduction

The physical and mental health of a population is directly and indirectly affected by the environment – for better or worse [1]. Whereas the probability of facing health risks due to cold weather is decreasing as the climate changes, extreme weather events, such as storms, heat waves and floods, are having direct negative consequences on health [1]. Changes indirectly affect the environment, and among the ways they do include the occurrence of infectious diseases and allergies [1]. At the center of planetary health as a concept is the interdependence between the natural environment and human health, with the overall aim of maintaining healthy people on a healthy planet [2].

The impacts of environmental changes on human health as a consequence of the climate crisis are becoming increasingly visible in Germany, also [2]. Our healthcare system faces the new challenges of adapting to the consequences of climate change, actively contributing to climate neutrality, seeing that healthcare professionals collectively acquire competency, and changing the way in which healthcare is delivered as a direct response to climate change [3], [4].

Alone the constant confrontation with the negative effects of climate change can give rise to anxiety, fear, distress and symptoms of depression [1]. Moreover, heat waves cause numerous fatalities primarily among the elderly due to their decreased thermophysiological ability to adjust [1], [5]. All of the examples presented here thus far make it clear that planetary health needs to be integrated into almost all of the subjects in the existing medical curricula. The teaching materials needed for this are available, primarily in digital form but without reference to the curriculum. Planetary health is a paradigmatic example for the necessary adaptation of the healthcare system and, correspondingly, of medical education.

The CanMEDS role of the “scholar” accompanies physicians over the entire course of their medical careers. It entails not only life-long learning, research and evidence-informed reasoning, but also the key concept of the “teacher” [6]. This role can be assumed for the first time during medical studies [7], in that students are duly trained and deployed as peer tutors in medical education. An early and trans-institutional qualification of students in medical education in the form of basic tutor qualification has been lacking thus far. Tutor training is usually limited to a specific location and often applies only to a specific medical school [8].

Students are also diversely engaged in the continuing development of education at medical schools and at the national level in the German Medical Students' Association (Bundesvertretung der Medizinstudierenden in Deutschland e.V., abbreviated as bvmd) [7]. Students take on an important role here as "change agents" in order to advance innovations in education [9], [10].

In large sections of medical education there is a discrepancy between the current state of knowledge in education research and its transfer into practice [11]. In a survey of members of the Association for Medical Education in Europe (AMEE), over 50% of the respondents indicated that their colleagues would not be very willing to change their teaching practices [12]. Frenk et al. describe the formation of networks and alliances, as well as the creation and benefits of shared learning resources, as important factors in the development of transformative, interdependent education that adequately responds to the need for changes in the healthcare system and, at the same time, induces needed changes [13].

Since its establishment in 2004 at the University of Heidelberg, the Master of Medical Education (MME) study program strives for the professionalization of multiplicators and leaders in education [14]. The program’s inter-faculty orientation, strong alumni network, and ongoing adaptation of its study program to reflect the needs in medical education have resulted in a strengthening of research efforts, mutually beneficial use of resources, and an emphasis on the visibility and professionalization of academic teaching at medical schools [14].

According our knowledge, there has been no other qualification measure that connects a trans-institutional graduate study program with a corresponding trans-institutional undergraduate program.

The project's aims encompass:

1. Supporting the *implementation of planetary health in the curricula* of the healthcare professions in Germany through the use of change agents at all levels;
2. *Networking between medical faculties* to connect committed medical students and multiplicators in academic teaching and to form a *community of practice* consisting of change agents revolving around planetary health and medical education;
3. Developing an *innovative elective subject* that qualifies *medical students as teachers* in a professionally oriented manner;
4. *Professionalization* of MME students in *digital teaching*.

## 2. Project description

The development of the elective course “Planetary Health in Medical Education” (ME elective) is presented below and follows the six steps of curriculum development as defined by Kern et al. [15].

During the MME module and the teachers' conference in 2021, new challenges were identified and worked through by the study program leadership, teachers and MME students, with the participation of students active in bvmd. Here it became evident that the MME study program can represent a joint platform to generate prototypical materials on planetary health, reinforce students' knowledge and communication skills, and boost the professionalization of teachers in the area of digital education.

The ME elective was developed in cooperation with the MME and based on prior work of bvmd and synergetically linked to the MME study program.

### 2.1. Step 1: Problem identification and general needs assessment

Planetary health is not a firmly established subject at present but is nonetheless relevant to all medical students regardless of semester level or specialty interest: The subject relevance of planetary health ranges from general practice (e.g., climate-informed medical advice) to surgery (e.g., nutrition-based etiology of colon carcinomas, gallstones and diverticulitis) and integrative medicine (e.g., resource-based medicine) to pediatrics (e.g., increase in allergies) and dentistry (e.g., microplastics and changes to the structure of tooth enamel) [16].

In the role of near-peer teacher, students not only solidify their knowledge and gain didactic skills [17], [18], but also express their desire to be included more in education in the future [19]. However, apart from tutor training programs, there is often no opportunity to acquire such qualification for students who are interested in topics beyond practical teaching such as, for instance, curriculum development, assessments and evaluation, or career development in education.

To use the advantages and benefits of digital education – including the promotion of planetary health through the reduction of emissions by not commuting [20] – as extensively as possible requires the take up of training in digital formats for students, peer teachers and instructors [21].

#### 2.2. Step 2: Targeted needs assessment

Courses on planetary health that are longitudinally anchored in a medical curriculum are currently scarce [5]. Calls have been made to anchor planetary health in the curricula for undergraduate, graduate and post-licensure education [3], [4]. There is interest and awareness of the issue among medical students internationally [20]. Yet, a professional sense of responsibility as future doctors in relation to patients and society is less pronounced [22].

**Figure 1:**
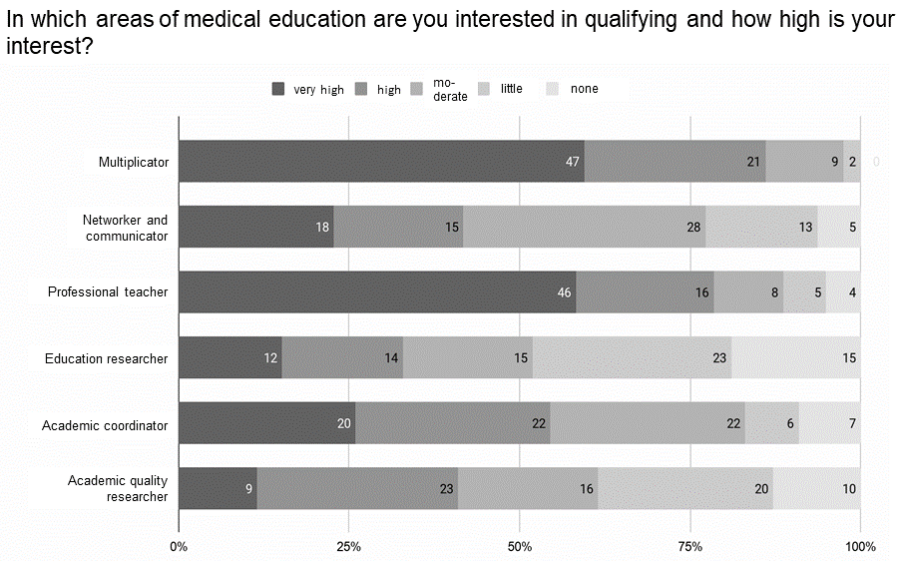
Qualification interests of the students according to areas in medical education (n=80)

The targeted assessment of medical students' needs regarding aspects of medical education was carried out using a survey and in a student workshop held by bvmd. The majority of the students who responded to the survey (n=80, response rate 8%) stated that they had very high or high levels of interest in the role of multiplicator (n=68, 85%) and the role of the professional teacher (n=62, 78%) (see figure 1 (Fig. 1)). The topics of interest mentioned most frequently by the students were “practical teaching and instructing” (n=46, 58%), “fostering the motivation to learn, creating a learning atmosphere” (n=34, 43%), “life-long learning and the principles of learning theory”, “giving feedback, training communication skills”, and “careers in medical education” (each with n=28, 35%). Other needs were expressed in the subsequent online workshop to design a course on medical education for students, including the use and integration of existing resources (teaching videos, presentation slides, etc.), inclusion of students in the ME elective, and application of the flipped classroom concept.

From the evaluation results and the surveys pertaining to the MME study program, it became clear that the MME students and teachers desired more digitalization of the study program components [23]. Moreover, “digital teaching” is a stated objective of the MME study program and implemented methodically by way of current digital teaching formats and in terms of a multiplicator concept.

#### 2.3. Step 3: Learning goals and objectives

The learning objectives listed in the National Catalogue of Learning Objectives in Undergraduate Medical Education (NKLM) 2.0 regarding planetary health [24] offer students an ideal starting point for planning and conducting their own seminar sessions on this topic. Content-based learning objectives were taken from this list, e.g., considering the increased risk of dehydration in elderly patients during heat waves caused by anthropogenic climate change and identifying suitable treatments. Additional learning objectives include being able to advise patients on immunization and name the basic aspects of the planetary health diet.

The main learning objectives regarding the role of the scholar are documented in the NKLM 2.0 (see Competencies VIII.1-05, [https://www.nklm.de]). In reference to it, students can rate, among other things, methods and strategies for teaching, learning and evaluation in adult education, as well as procedures for assessment and evaluation, in terms of their strengths and weaknesses and the resources needed for them. Students also choose a specific learning situation.

The learning objectives regarding digital teaching in the MME study program entail, among other things, being able to prepare an online course aimed at a specific audience and to systematically collect course feedback online and analyze it.

Drawing up the specific learning objectives for each course session was done on the basis of a bvmd concept in collaboration with the MME students belonging to cohort 18 in the form of the follow-up work associated with module 3 “teaching and assessing 1” that focuses on teaching, learning, assessing and evaluating.

#### 2.4. Step 4: Educational strategies

A clinical elective subject was to be offered online in order to give students a trans-institutional option for a course on planetary health in medical education. The selected examples to support the teaching strategy were taken from the subject of planetary health so as to reach a wide range of students with a variety of interests.

Digital education includes different forms of teaching and learning; it is both effective and necessary in these pandemic times to enable continuous education for medical students [25]. The design of an online elective not only offers the chance to have a trans-institutional, geographically unbound course, but also to develop competency in digital teaching in students and teachers [26]. The concept takes into account the “zone of proximal development”, in which students can optimally develop their skills through being coached by the MME students and MME students through being supervised by the MME module instructors [27]. Students gather and develop their own resources in that they prepare a course session on a topic in planetary health. Because learning is an integral part of practice, actively including the students in the MME students’ classroom teaching and in the modules in the form of legitimate peripheral participation are meant to be the first steps into real practice for the students [28].

Since an elective course involves a limited period of time, this places example-based learning in the foreground [29]. The ME elective was designed accordingly with regard for the current state of educational science so that it, itself, could serve as a best-practice example for good teaching. This was achieved by using the flipped classroom model [30] with synchronous tutorial sessions and asynchronous preparatory assignments, the use of various didactic methods and the sandwich principle [31]. Digital tools were applied by the students in a reflective manner, in compliance with data protection rules, to support the development of the seminars according to the sandwich principle.

The concrete work to design the lesson plans and teaching strategies was done by the MME students in cohort 18 under constant supervision by the project team.

#### 2.5. Schritt 5: Implementation

The final concept for the ME elective encompassed 28 course units (UE), amounting to 2 hours per week per semester and consisting of 4 phases: introduction (8 UE), course planning by the undergraduate students with support from the MME students (10 UE), seminar sessions held by the undergraduate students under the supervision of the MME students (6 UE), and networking with the MME study program (4 UE). Planetary health as an interdisciplinary topic with interprofessional points of overlap has relevance regardless of semester level.

**Figure 2:**
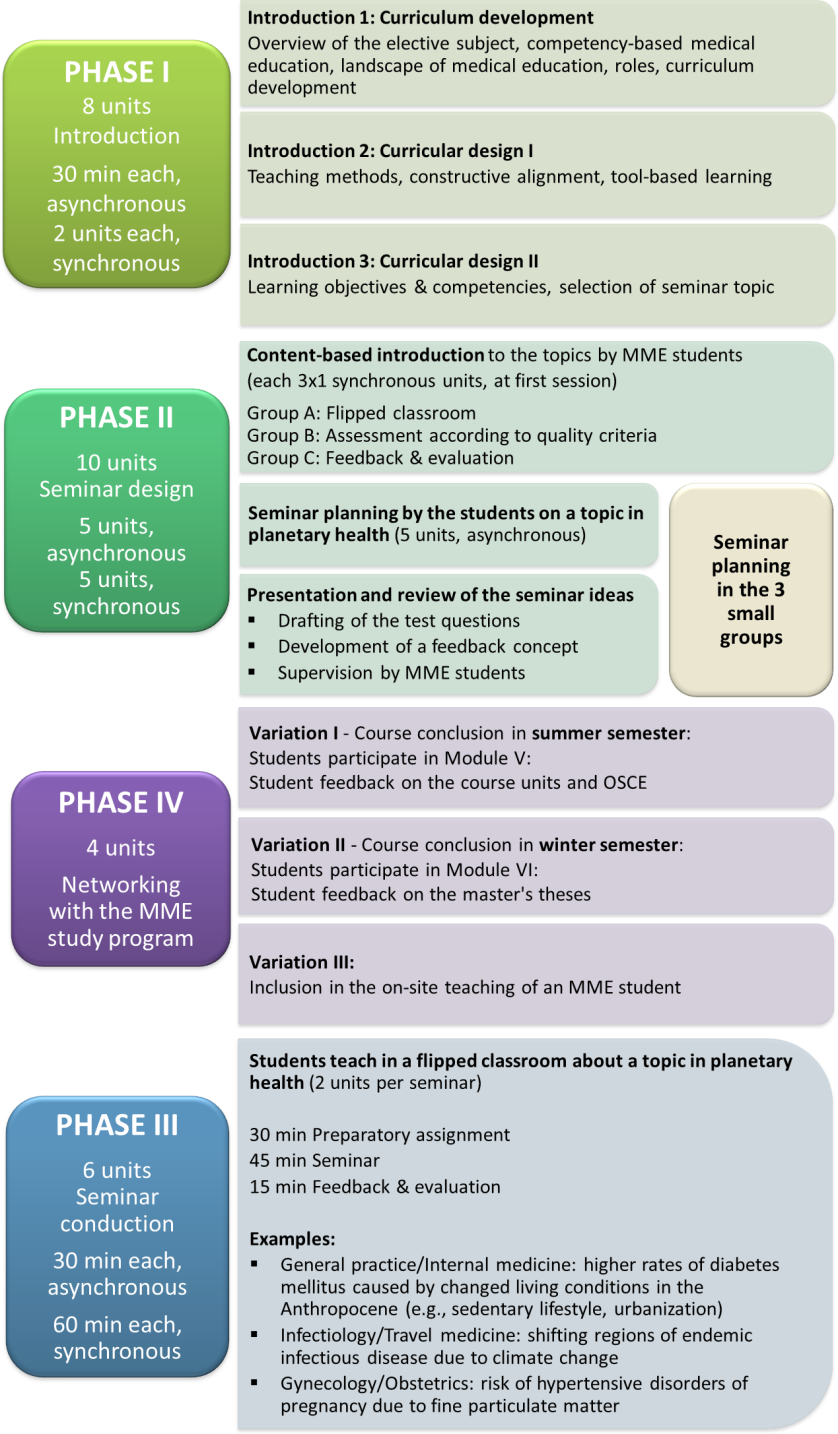
Detailed sequence of the Medical Education elective with phases I to IV

The detailed course sequence of the ME elective is illustrated in figure 2 (Fig. 2). The course sessions take place during the semester to implement the flipped classroom format and give the students and instructors a space in which to become acquainted with each other and to begin networking. The longitudinal use of feedback makes it possible to prompt and steer the ongoing development of competency, above all, in planetary health and digital teaching [32]. 

A total of 35 undergraduate students applied to attend the ME elective during its pilot in the 2022 summer semester. Twenty-four students from 13 medical schools – Dresden, LMU Munich, Tübingen, Cologne, Heidelberg, Aachen, Berlin, Halle-Wittenberg, Jena, Brandenburg, Oldenburg, Saarland, and Vienna – attended during the 2022 summer semester. Admission to the course was based on random selection.

#### 2.6. Step 6: Evaluation

A scientific evaluation was carried out on multiple levels simultaneously with the course according to Kirkpatrick's framework [33] and entailed, on the one hand, the evaluation by the participating undergraduates and the MME students in the role of instructor.

This evaluation is supplemented by an external appraisal by the project team and the attending students using an evaluation instrument that examines the course sessions planned and held by the students regarding sequencing, organization, topic choice, and the conduction of the seminar.

The program evaluation also evaluated the course units with questionnaires asking for assessments of the teaching method, the content, the opportunities to participate, and discussions among the participants.

The results of this concomitant evaluation enable adjustments to be made for the second round of the ME elective. The concomitant evaluation is designed, developed and analyzed within the scope of project papers and master's theses.

## 3. Discussion and critical reflection

The aim of the ME elective was to establish planetary health as a transformative educational topic across different medical schools and to include the topic of medical education in medical studies. For the MME students, the aim was to directly transfer what has been learned into practice and to reinforce competencies in digital teaching and the role of the multiplicator. For the medical education community this means promoting young doctors, professionalization, identity formation and networking, horizontally (trans-institutionally) and vertically (between the different semester levels of the undergraduates and MME students).

Within the larger topic of planetary health, the ME elective builds a bridge between the qualification of peer tutors and the qualification of university instructors at medical schools. This interdisciplinary and interprofessional topic is thus integrated into an innovative concept for trans-institutional and interlinked education that teaches the learning objectives contained in the NKLM. Maintaining the presence of planetary health as a subject requires collaborative work, and it appears suitable for sparking transformative learning among students in small groups. The inclusion of planetary health as a main topic also addresses the important leadership aspects in medical education [34]. Target groups are reached from all areas, since the participants involve not only medical students, but also MME students, who are frequently medical residents or already medical specialists. This approach represents an opportunity to strengthen the competence of medical professionals long term as they provide medical care for health problems caused by climate change.

Online courses often lack an opportunity to apply the acquired knowledge in practice. The Planetary Health Academy offers a publicly accessible lecture series [https://planetary-health-academy.de/de/programm/], just as the existing AMEE online course *Essential Skills in Medical Education Students* does, with an opportunity to participate for medical students and residents from all over the world [https://www.esmecourses.org/course/index.php]. However, with the ME elective the application of knowledge regarding planetary health and medical education is extensively supported in a structured and supervisory manner as a result of designing and holding course sessions and participating in university teaching during phase IV of the ME elective. At the same time, the ME elective supports the start of transformative action [13] at the medical schools.

The need among students for courses on medical education which was identified in the needs assessment was confirmed in the high numbers of course registrations within a short period of time. Students who enroll in the ME elective are introduced early on to the landscape of medical education and can build on their competencies in the area of teaching. This includes the acquisition of competency in the imparting of content which the students themselves have not yet encountered or have barely encountered in their previous coursework and as such poses increased demands on them in the role of teacher. This enables them to perform their work professionally in formal student bodies or as tutors.

The extent to which the ME elective is able to expand and deepen the digital competencies of the participating MME students in the role of teacher will be shown in the concomitant evaluation. Likewise, direct feedback from students regarding the online seminar can actively support personal development and growth.

Generally, an elective course that is not limited to one university offers great potential regarding the augmentation of subjects and topics at many medical schools. For this reason, the recognition of such trans-institutional elective subjects at the individual universities is worth striving for and, although it means more effort in terms of organization than a university's own elective courses do, it also poses great innovative potential with generous leeway to specifically cover the students' range of interests.

## 4. Conclusion

Planetary health unifies interests spanning a wide range of subjects and semester levels and lends itself as a collaborative, interdisciplinary and interprofessional endeavor to rally students around a common cause. The undergraduate medical students and the MME students were able to serve as multiplicators to introduce planetary health at their own medical schools in their capacity as change agents.

The ME elective contributes to the horizontal and vertical networking of students and instructors across university medical schools in the area of planetary health and transformative medical education. It empowers students to plan their own course sessions on planetary health and to teach them in a virtual flipped classroom.

## Author contributions

Kristina Flägel, Matthis Manke and Katharina Zimmermann contributed equally to this work and share joint first authorship.

## Competing interests

The authors declare that they have no competing interests.
